# Defining Health in the Era of Value-based Care: Lessons from England of Relevance to Other Health Systems

**DOI:** 10.7759/cureus.1079

**Published:** 2017-03-06

**Authors:** Sarah Gentry, Padmanabhan Badrinath

**Affiliations:** 1 Directorate of Public Health and Protection, Suffolk County Council

**Keywords:** value-based healthcare, health service, quality, public health

## Abstract

The demand for healthcare is rising due to aging populations, rising chronic disease prevalence, and technological innovations. There are currently more effective and cost-effective interventions available than can be afforded within limited budgets. A new way of thinking about the optimal use of resources is needed. Ensuring that available resources are used for interventions that provide outcomes that patient’s most value, rather than a focus just on effectiveness and cost-effectiveness, may help to ensure that resources are used optimally. Value-based healthcare puts what patients value at the center of healthcare. It helps ensure that they receive the care that can provide them with outcomes they think are important and that limited resources are focused on high-value interventions. In order to do this, we need flexible definitions of ‘health’, personalized and tailored to patient values.

We review the current status of value-based health care in England and identify lessons applicable to a variety of health systems. For this, we draw upon the work of the National Institute for Health and Care Excellence (NICE), the National Health Service (NHS), Right Care Initiative, and our local experience in promoting value-based health care for specific conditions in our region. Combining the best available evidence with open and honest dialogue between patients, clinicians, and others, whilst requiring considerable time and resources are essential to building a consensus around the value that allows the best use of limited budgets.

Values have been present in healthcare since its beginnings. Placing value and values at the center of healthcare could help to ensure available resources are used to provide the greatest possible benefit to patients.

## Introduction and background

Demand for healthcare is rising in many countries around the world due to aging populations, the rising prevalence of chronic disease, and improving medical technology [1]. With limited healthcare budgets, it is important that resources are used in a way that provides the most value for patients.

There has been acknowledgment for many years that health is much more than just the provision of healthcare. The World Health Organization defines health as ‘a state of complete physical, mental, and social well-being and not merely the absence of disease or infirmity’ [2]. Improving health includes broader aims, such as tackling the social determinants of health, allowing people the ‘freedom to lead lives they have reason to value’ [3]. The value-based care movement involves greater recognition of what patient's value when defining health and care outcomes and allocating limited resources. This review aims to give an overview of value-based healthcare, the advantages and disadvantages and how it can be applied, and then to discuss the important challenge of defining health within this framework. We share examples of how we defined health in the context of value locally with respect to patellar resurfacing during knee replacement, carpal tunnel decompression, and tonsillectomy. By using evidence and reaching local consensus through consultation as a local health system, we agree when an intervention adds value to both the patient and the system.

## Review

### What is value

There are many different definitions of value and value can be considered from many perspectives including that of the payer (government, insurance company, or individual), clinician, healthcare industry, caregivers and most importantly, the patient [4]. Definitions can also be considered from the perspective of the public and private sectors [5]. Some definitions of value focus on the moral aspects, such as principles, standards, and importance. Others focus on the economic meaning, such as the definition proposed by Porter that ‘Value = outcomes achieved – money spent’ [6]. The definition proposed by Gray covers aspects of each of these and defines value in healthcare as ‘the net benefit, that is the difference between the benefit and the harm done by a service, taking into account the amount of resources invested’ [4]. He then goes on to subdivide value into three types [7]:

1. Allocative value – how to allocate resources equitably in such a way that maximum value for the whole population is obtained 2. Technical value – increased value associated with improvements in quality and safety of healthcare 3, Personalised value – individual patient values, in combination with best evidence and assessments of the person’s condition.

The importance of providing value, as opposed to just cost-effectiveness, effectiveness, or efficiency, is increasingly being considered of importance and delivering value is one of The King's Funds priorities for the United Kingdom (UK) National Health Service (NHS) and social care services in 2017 [8].

### How could value-based healthcare work?

Value-based healthcare is a way of trying to ensure that limited resources are used in a way which provides the greatest value to patients. With the growth in new technology and treatments, the aging population and growing burden of chronic disease many countries are not able to fund unlimited healthcare through their current budgets. One way around this would be to increase health care funding, but this requires taking money away from other areas, which for most people, would only be acceptable up to a point. Archie Cochrane famously reflected in 1972 that ‘All effective treatment must be free’ [9]. However, it may now be that due to advances in health technology, there are more effective interventions available than can be afforded [4]. Even if it is affordable, it may not be valuable. Unlimited healthcare intervention provision may lead to increased harm. Donabedian suggested that there is a law of diminishing returns in health care, with greater investment leading to better outcomes up to a point, at which greater resource input leads to limited improvement in outcomes [10]. He also argued that healthcare-associated harm was directly correlated with the amount of healthcare provided. These two ideas combined suggest that there is a point at which greater investment would actually lead only to more intervention associated harm, with very little improvement in outcomes. Gray suggests that maximum value is the point at which investment of resources is providing maximum benefit and has not yet approached the level beyond which increased investment would lead only to greater health intervention associated harm [4].

One of the roles of The National Institute for Health and Care Excellence (NICE) in England is to assess the cost-effectiveness of interventions, and only recommend for funding those under a certain threshold [1]. However, with such rapid innovation in medical technology, rather than just more effective interventions available than can be afforded, there are also more interventions that have been shown to be cost-effective than can be funded from current health budgets [4]. There are also many variations in healthcare provision within and between countries in terms of services accessed and outcomes obtained. For example, the percentage of people who have had an acute stroke who received thrombolysis in England in 2014 varied from 2.8% (95% confidence interval 0.9 to 7.9) in Corby to 32.7% (95% confidence interval 25.7 to 40.5) in Ashford [11]. Most of this variation cannot be explained by differences in patient need or choice and thus is considered unwarranted. Alderwich, et al. suggest such unwarranted variation indicates that resources in some areas may be being overused and underused in others [12].

Whilst effectiveness, cost-effectiveness, and quality are of great importance, it is possible for an intervention to be all of the above, but still of limited value for an individual patient. For example, Gray suggests that recommending heart surgery to an older patient whose main concern is becoming tired after two hours gardening may involve the provision of an evidence-based, cost-effective and high-quality treatment, which still does not give the patient, the outcome they value. Provision of interventions or care to patients that do not add value from the patient's perspective is an example of overuse and may lead to more harm than good, as all healthcare is associated with some amount of risk [4]. Underuse also reduces value, as patients do not receive care that adds value to their lives and may lead to greater cost down the line. A focus on funding procedures of highest value to patients can try to make the best use of limited resources and avoid waste through overuse of low-value interventions.

Some have suggested that value-based healthcare could revolutionize the way in which healthcare is managed and begin to tackle some of the key challenges facing health systems across the globe, whilst others suggest the benefits are more likely to be incremental, with small changes made to services and systems over time [12].

### Managing demand

Public health interventions play a key role in managing need by making populations healthier, but initiatives with this aim often take many years to have a significant impact. Value-based healthcare cannot reduce need but it does have the potential to manage demand by clarifying which subgroups are most likely to benefit from an intervention, ensure patients are aware of the potential benefits and harms of interventions and manage innovations to ensure they are not only effective but provide value, to ensure limited resources are used for the greatest patient benefit [4].

### Appling values to evidence-based medicine

Evidence-based medicine, which can be defined as ‘the conscientious, explicit and judicious use of current best evidence in making decisions about the care of individual patients [13], and the underpinning methodological rules and research, involve more than objective facts and include value-laden judgments [14]. Kelly, et al. argue that values ‘infuse evidence at many levels’, not just at the point of care decisions with patients and their clinicians. The questions asked by the objective researcher, and the methods they use to try to answer these questions are laden with often unacknowledged values. Acknowledging and embracing the presence of values reduces hidden bias, adds meanings to findings and encourages societal discussions about which values should drive resource allocation [14].

### Of relevance in a variety of health systems

Value-based healthcare involves all stakeholders accepting ‘accountability for improving patient outcomes relative to their cost’ [15]. How these stakeholders are organised varies hugely between countries, but value-based healthcare can be applied in different ways in different health systems.

Much of the work done on value-based healthcare focusses on its role in health systems in which, the value providers are separate from those who pay for healthcare (whether funded by taxation, compulsory social insurance or individual or employed-based insurance) [6]. In England, since the 2012 Health and Social Care Act, local Clinical Commissioning Groups (CCGs) made up of general practitioners, other clinicians and lay members, have been responsible for planning, designing and buying local health services from both public and private providers using government funding [1]. Value-based health outcomes assessments can be used as part of the payment of health providers in health systems with a purchaser-provider split. Porter argues this would promote effective competition and improve value [6]. To accurately gauge value in is, he argues value needs to be estimated based on an entire ‘cycle of care’ and payments to providers made in bundles, rather than payment for procedures, payment for following processes or guidelines or global capitation (payment per person/per time period) [16-17]. He argues this could allow interventions with high value which lead to future cost savings to be incentivised and rewarded.

There are also many opportunities to apply value-based healthcare for the benefit of patients in health systems without a purchaser-provider split, such as the Scottish and Welsh health systems. Value based outcome assessment can be used to establish which services are of high value and identify areas of overuse and waste, ensuring resources are used in a way which maximises value for patients and reduces harm. Such a form of assessment may also help change the focus of the provision of healthcare from what healthcare professionals do to what patients value [6].

Where patients must pay ‘out-of-pocket’ for healthcare, including most low- and middle-income countries, assessment of value may still be possible, but it is unlikely that such assessments will be sufficient to promote change and would be challenging to implement.

Where used, value-based healthcare has the potential to enable more accurate comparisons between services in different regions and countries.

In the moral sense of the definition of value, decisions by governments about how a health system should be structured and what proportion of gross domestic product should be spent on healthcare as opposed to something else is very much a value based choice [4].

### Pay for performance in England

A variety of pay-for-performance measures has been tried in the English National Health Service (NHS) [1]. The Quality and Outcomes Framework (QOF) is an incentives programme for primary care in which general practice (GP) surgeries are rewarded financially for providing quality of care for chronic diseases such as asthma and diabetes, health behaviours, such as smoking and obesity, and implementation of preventative healthcare measures such as blood pressure checks. Best Practice Tariffs (BPTs) are used in secondary care to provide financial incentives to encourage achievement of criteria thought to improve quality and cost-effectiveness. For example, a BPT relating to fragility fractures of the hip rewards hospitals with short waiting times for operations and early involvement of orthogeriatricians [18]. Another example is Commissioning for Quality and Innovation (CQUIN), a tariff system set up by the Department of Health in 2009. It allows health commissioners to ‘hold back 2.5% of the cost of hospital treatment contingent on outcomes’ [1]. Such models could also be used for state-of-the-art treatments, with a proportion of the payment given only after efficacy and safety have been shown. However, assessing the effectiveness of such methods is challenging and the available evidence is limited [1].

### Moving from cost-effectiveness and pay for performance to values-based health care

Reducing the amount of money spent on a health service without considering value can lead to false economies and be self-defeating [16]. With a focus on achieving the most value for patients, rather than focussing on cost reduction or efficiency, it is hoped that healthcare professionals, managers, and other stakeholders may be able to work towards a common aim of providing value in healthcare [15-16].

One example of a move toward value-based outcome assessment in the English NHS is Patient Reported Outcome Measures (PROMs) [19]. These aim to assess the quality of care from the patient perspective, initially for four common clinical procedures (hip replacement, knee replacement, groin hernia and varicose veins), before moving onto common chronic diseases. PROMs calculate the health gains from treatment using pre- and post-intervention surveys. Data from this assessment is published, but the results are not used in deciding payment to healthcare providers. Another example is the Friends and Family Test (FFT), an important feedback tool that supports the fundamental principle that people who use NHS services should have the opportunity to provide feedback on their experience. It asks people if they would recommend the services they have used and offer a range of responses. When combined with supplementary follow-up questions, the FFT provides a mechanism to highlight both good and poor patient experience. This kind of feedback is vital in transforming NHS services and supporting patient choice.

The ‘Realising the Value’ programme funded by NHS England and led by the Health Foundation and Nesta highlighted how person- and community-centred approaches, such as peer support, self-management education, health coaching, group activities and asset-based approaches could be used to promote health and wellbeing, and add value to the lives of individuals and communities whilst remaining financially sustainable [20]. They recommend trials of person- and community-centred payment methods, learning from different payment methods currently used the NHS and trials to inform payment method reform.

Value-based pricing, in which the price paid to pharmaceutical companies for drugs is based on evidence of value for patients, as opposed to cost-benefit calculations, could incorporate wider factors such as benefits to society and could improve access and encourage innovation, but more rigorous evaluation is needed [21].

Value-based healthcare has the potential to be used in local and national priority setting and policy development. The value should also be considered more broadly than just within healthcare services, encompassing public health and local government to tackle wider determinants of health. According to a recent publication by the Institute of Healthcare Management, system leadership is essential for the survival of the health service [22]. Similarly, taking steps to define, develop and implement value-based health care could help health systems swim rather than sink and allow them to continue to provide the services upon which millions of citizens depend.

### Tools for implementing value-based healthcare

A variety of tools has been developed for use in England to help the NHS to deliver value.

The NHS RightCare programme aims to offer better value for patients by ensuring they receive the right care at the right time and place, focussing on areas with the greatest opportunity to improve health and make sustainable improvements to reduce unwarranted variation [23]. Part of this is the Shared Decision Making (SDM) program, which aims to support patients in taking control of their health, encouraging patient centred care, choice, and autonomy, by supporting SDM, the conversation between a patient and their healthcare professional which leads to a joint decision about their care. To ensure patients are placed at the centre of care and able to control their health, we must provide them with the tools in which to do this. Achieving such a transformation is complex and must consider patient’s individual circumstances, contexts, and cultures. 

To support CCGs in delivering value-based health care, they are provided with ‘Commissioning for Value’ packs allowing them to compare indicators of how they are doing in different aspects of treatment pathways for different conditions compared with CCGs with similar characteristics. This aims to support them in identifying areas of unwarranted variation and areas for improvement with greatest potential for increased value [24]. Casebooks are also available with examples of best practice to assist CCGs with how to improve value in the areas identified through their value packs. One such casebook gives the example of how data can be mapped to each stage of the diabetes care pathway and used to identify areas of unwarranted variation in outcome, leading to the identification of areas for improvement that may have been missed under traditional methods of monitoring based on process measures [25]. CCGs are encouraged to compare their outcomes with other areas with similar demographic characteristics and to seek advice from those providing better value for their populations. Such data can be used to improve value for both individual patients and for populations through optimum resource allocation.

### Local examples of defining value 

Local purchasers of care, operate a priority setting process and mechanism to ensure that the limited health care resources are spent in the best possible way to maximize health gain. We provide three conditions as examples of how the concept of value in health care was considered and adopted. The local payers were spending many hundreds of thousands of pounds on ‘patellar resurfacing’ during knee replacement. As one of the local hospitals was an outlier, questions were raised about the value of this intervention. We undertook a thorough review of the evidence and found that evidence for the intervention was lacking. We realized the opportunity cost of the intervention and undertook a consensus building exercise with patients, managers, clinicians and the public on the value of patellar resurfacing. At the end of the consultation period, the system arrived at a consensus that it does not offer value and hence should be disinvested. 

Carpal tunnel decompression rates were significantly higher in one of the local institutions compared to the national and regional rates after standardization. A similar approach was adopted and a detailed pathway and access criteria were developed which reduced the rates to a significant extent. It was apparent that there was overuse of the procedure and the value to patients and the system was being ignored. Tonsillectomy rates show huge variation across the country and this has also been addressed in a similar manner. Having an open and honest dialogue with both patients and clinicians and their support is essential to promote values-based health care. It is important to avoid the perception that this is simply a cost cutting measure and considerable time and resources need to be deployed to build a consensus around value and the approach to take to achieve this.

### Measurement of outcomes

Measuring whether or not the value is achieved for patients requires comparable outcome measures based on what patients value [15]. Porter recommends classification of outcomes in three tiers [16]. Tier one is ‘Health status achieved or retained’, including measures such as survival at one or five years, or for those with life-limiting conditions, the degree of health or recovery achieved or maintained. Tier two, ‘Process of recovery’, includes the time taken to return to normal activities and disutility of care, such as errors and adverse events in care, incorrect diagnosis, and discomfort. Tier three is ‘Sustainability of health’ and includes recurrence and long-term consequences of treatment. 

The International Consortium for Health Outcomes Measurement (ICHOM) is a non-profit organization using a theoretical framework developed by Porter and Teisberg [26] to define global outcome standard sets based on what matters to patients and then driving adoption and reporting of these measures across the world [27]. Their outcome sets include common diseases, such us cancers and cardiovascular disease and primary and preventative care for older people. They report they have published standard sets covering 47% of the global disease burden and aim to increase this to over 50% in 2017.

### Defining health

The idea of health as the absence of disease has long been considered insufficiently broad and not covering all aspects of health. The World Health Organization definition of health presented in the introduction to this review is aspirational, recognizes all aspects of health and its effects and incorporates a subjective element. Unfortunately, it is at present likely unobtainable for many, particularly with the rise in chronic disease and an aging population [28], with many people with multi-morbidity and frailty. With its focus on the ‘complete,’ some suggest there is the risk of over-medicalization, at it suggests any slight deviation from complete health being an indicator that care may be needed, reducing autonomy. It is also hard to measure and is not tailored to the individual. It is, therefore, perhaps of limited use for assessing whether a health system is providing value to patients.

Greater acknowledgment of areas of wellbeing providing wider value to older patients and those with chronic illnesses, such as societal participation and coping capacity, may allow for a more practical and thorough assessment of value in healthcare, particularly in high-income countries. Huber, et al. suggested that perhaps a definition of health focussed on ‘the ability to adapt and to self-manage’ may be more appropriate for today's society [28]. However, in narrowing the definition of health, the suffering associated with even a well-managed chronic illness may be neglected [29]. Shilton, et al. emphasize the importance of health as a human right and the effects of social, political, economic and environmental factors, particularly with the wide health inequalities present within and between countries and the need to tackle the wider determinants of health [30]. They propose a definition of health focussed on giving people the income, education, and power to control their lives, highlighting the role of supportive systems, environments, and policies in enabling health. Value-based health care is one way of trying to put patients at the center of healthcare and give them the freedom to control their health. For value-based healthcare to become truly influential, a focus on value would need to reach more broadly than health services alone.

Our vision of health is that any individual in society is able to live the best life possible according to what they value, facilitated by access to high quality, patient-centred, evidence-based and affordable health and care services and supported by a strong public health system. We feel taking a value-based approach will help to achieve this.

To develop a definition of health that is useful for the implementation and assessment of value-based healthcare, the suggestion by Huber, et al., that different definition be developed for different purposes and contexts, with objective and subjective measures as appropriate, may be practicable [28]. It is important that definitions used are validated for the cultural setting in which they are applied.

## Conclusions

Values have been present in healthcare since its beginning. Placing value and values at the center of healthcare could help to ensure, available resources are used to provide the greatest possible benefit to patients.

With an aging population, the greater burden of chronic disease and the development of new technologies, demand for healthcare is increasing, whilst healthcare budgets are limited. How much of a societies resources should be used in healthcare is a value based judgment. Ensuring that available resources are used for interventions that provide outcomes that patients most value, rather than a focus just on effectiveness and cost-effectiveness may help to ensure that resources are used optimally.
